# Biopersistence of NiO and TiO_2_ Nanoparticles Following Intratracheal Instillation and Inhalation

**DOI:** 10.3390/ijms18122757

**Published:** 2017-12-19

**Authors:** Takako Oyabu, Toshihiko Myojo, Byeong-Woo Lee, Takami Okada, Hiroto Izumi, Yukiko Yoshiura, Taisuke Tomonaga, Yun-Shan Li, Kazuaki Kawai, Manabu Shimada, Masaru Kubo, Kazuhiro Yamamoto, Kenji Kawaguchi, Takeshi Sasaki, Yasuo Morimoto

**Affiliations:** 1 Institute of Industrial Ecological Sciences, University of Occupational and Environmental Health, 1-1 Iseigaoka, Yahata-nishi-ku, Kitakyushu, Fukuoka 807-8555, Japan; tmyojo@med.uoeh-u.ac.jp (T.M.); leebw401@med.uoeh-u.ac.jp (B.-W.L.); okadatakami@med.uoeh-u.ac.jp (T.O.); h-izumi@med.uoeh-u.ac.jp (H.I.); y-yoshiura@med.uoeh-u.ac.jp (Y.Y.); t-tomonaga@med.uoeh-u.ac.jp (T.T.); liyunsha@med.uoeh-u.ac.jp (Y.-S.L.); kkawai@med.uoeh-u.ac.jp (K.K.); yasuom@med.uoeh-u.ac.jp (Y.M.); 2 Department of Chemical Engineering, Hiroshima University, Higashi-Hiroshima 739-8528, Japan; smd@hiroshima-u.ac.jp (M.S.); mkubo@hiroshima-u.ac.jp (M.K.); 3 National Institute of Advanced Industrial Science and Technology (AIST), 1-1-1 Higashi, Tsukuba, Ibaraki 305-8565, Japan; k-yamamoto@aist.go.jp (K.Y.); k-kawaguchi@aist.go.jp (K.K.); takeshi.sasaki@aist.go.jp (T.S.)

**Keywords:** NiO, TiO_2_, nanoparticles, biopersistence, lung burden, inhalation, intratracheal instillation

## Abstract

The hazards of various types of nanoparticles with high functionality have not been fully assessed. We investigated the usefulness of biopersistence as a hazard indicator of nanoparticles by performing inhalation and intratracheal instillation studies and comparing the biopersistence of two nanoparticles with different toxicities: NiO and TiO_2_ nanoparticles with high and low toxicity among nanoparticles, respectively. In the 4-week inhalation studies, the average exposure concentrations were 0.32 and 1.65 mg/m^3^ for NiO, and 0.50 and 1.84 mg/m^3^ for TiO_2_. In the instillation studies, 0.2 and 1.0 mg of NiO nanoparticles and 0.2, 0.36, and 1.0 mg of TiO_2_ were dispersed in 0.4 mL water and instilled to rats. After the exposure, the lung burden in each of five rats was determined by Inductively Coupled Plasma-Atomic Emission Spectrometer (ICP-AES) from 3 days to 3 months for inhalation studies and to 6 months for instillation studies. In both the inhalation and instillation studies, NiO nanoparticles persisted for longer in the lung compared with TiO_2_ nanoparticles, and the calculated biological half times (BHTs) of the NiO nanoparticles was longer than that of the TiO_2_ nanoparticles. Biopersistence also correlated with histopathological changes, inflammatory response, and other biomarkers in bronchoalveolar lavage fluid (BALF) after the exposure to nanoparticles. These results suggested that the biopersistence is a good indicator of the hazards of nanoparticles.

## 1. Introduction

Thanks to their various functions and extreme usefulness as industrial products, nanomaterials (TiO_2_, silica, carbon black, and carbon nanotubes, etc.) are being developed and manufactured in a wide variety of fields, including plastics, colorants, coating, cosmetics, semiconductors, and drug delivery systems. However, there are reports of their higher toxicity compared with micron-size particles and the possibility of the translocation of inhaled nanoparticles from the lung to the brain [1,2,3,4,5,6], although there are also differing results [7]. Indeed, the toxicity of nanomaterials—which have varying characteristics—has not been fully evaluated. There is an urgent need to evaluate the toxicity of nanomaterials, given the fact that numerous nanomaterials already exist, and expanded manufacture and use of nanoparticles is predicted.

As for the hazard indicator, several toxicity biomarkers, including pulmonary inflammation, polymorphonuclear neutrophils in bronchoalveolar lavage fluid (BALF), cytokine release, oxidative stress, and the biopersistence of particles in the lung, have been examined in inhalation exposure tests using animal models [8,9,10]. Among them, the biopersistence of inhaled particles in the lung is reported to be a useful toxicity index [11,12,13]. Biopersistence represents the level of difficulty in clearing particulate matter that has entered the lungs, and is usually shown as a biological half time (BHT)—that is, the time required for the level of particulate matter to reduce to half the initial level in the lung. Inhalation of toxic particles damages pulmonary scavenger cells such as macrophages, resulting in delayed pulmonary clearance of the particulate matter. Generally, slower or faster pulmonary clearance indicates higher or lower toxicity, respectively.

The toxicities of asbestos and its alternatives (man-made vitreous fibers) have been evaluated using the inhalation exposure test and the intratracheal instillation test: fibrosis and tumor development became more frequent as the pulmonary persistence of fibers with a length of ≥20 μm increased, indicating a correlation between biopersistence (BHT) and pathological changes [14]. Based on these findings, the pulmonary persistence revealed by in vivo tests has become the standard index for judging the toxicity of asbestos alternatives in the European Community directive relating to the classification, packaging, and labeling of asbestos alternatives. We have reported the biopersistence of various micron-size fibers and particles, and biopersistence had a good correlation with the pathological changes [15,16,17,18,19,20,21,22,23].

The biopersistence is a useful toxicity indicator for mineral fibers and micron-size particles. However, there is a possibility that the biopersistence of nanoparticles is different from these particles because of the translocation caused by the small size or the high solubility caused by the large surface area. Therefore, the investigation about whether the biopersistence is also available for evaluating the toxicity of nanoparticles is important and meaningful. Although we have reported the biopersistence of nanomaterials in inhalation and instillation [24,25,26,27] and there are some recent reports of nanomaterials [28,29,30,31,32], there is no report which verifies the usefulness or availability of biopersistence for evaluating the toxicity of nanoparticles.

The aim of this study is to determine whether biopersistence is a useful indicator for evaluating the toxicity of nanoparticles. To perform this task, we selected two nanoparticles with different toxicities—a highly toxic nanoparticle (NiO) and a low-toxicity nanoparticle (TiO_2_)—and performed inhalation and instillation studies at several doses and investigated whether or not each biopersistence is different, and furthermore whether the obtained biopersistence correlates to the histopathological changes and the other toxicity indicators.

## 2. Results

### 2.1. Biopersistence of NiO and TiO_2_ Nanoparticles in Inhalation and Intratracheal Instillation Studies

#### 2.1.1. Measured Amounts of Nanoparticles in Lung and Calculated Biological Half Time (BHT)

The measured amounts of nanoparticles in the rat lung are summarized in Table 1. Data are expressed in the form of mean ± STD.

**Figure 1:**
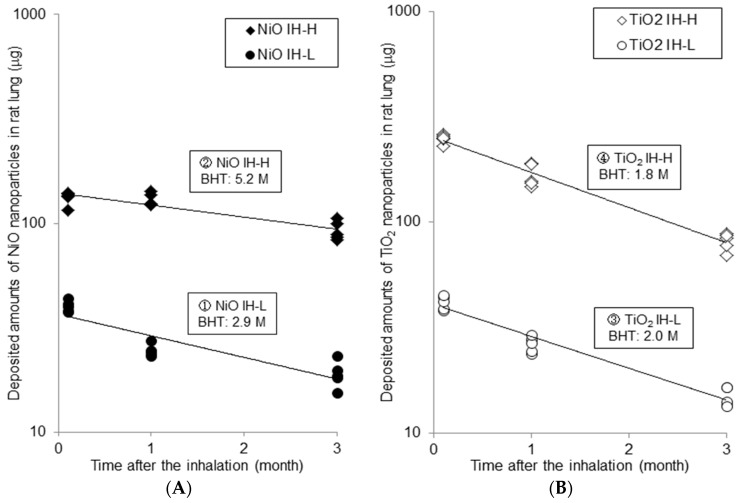
Biopersistence of NiO and TiO_2_ nanoparticles in rat lungs in inhalation studies. (**A**) NiO nanoparticles; (**B**) TiO_2_ nanoparticles.

The temporal change in the amounts of nanoparticles in the lung in the inhalation studies ((A) ① NiO-IH-L, ② NiO-IH-H (B) ③ TiO_2_-IH-L, ④ TiO_2_-IH-H) are shown in Figure 1.

**Figure 2:**
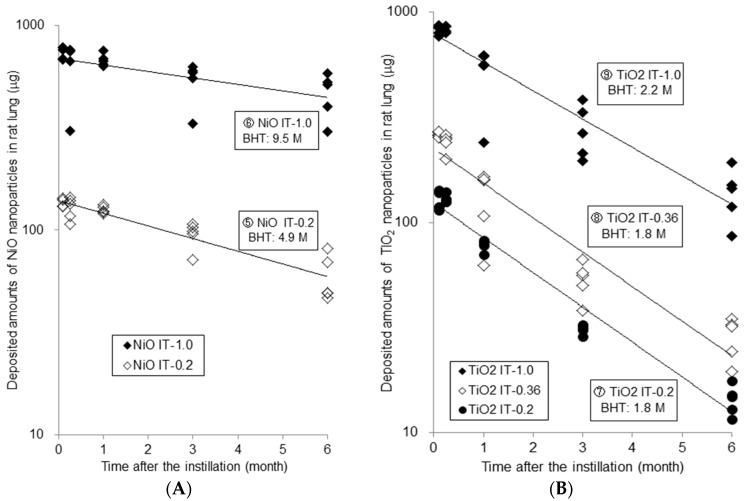
Biopersistence of NiO and TiO_2_ nanoparticles in rat lungs in intratracheal instillation studies. (**A**) NiO nanoparticles; (**B**) TiO_2_ nanoparticles.

Results of the instillation studies ((A) ⑤ NiO-IT-0.2, ⑥ NiO-IT-1.0; (B) ⑦ TiO_2_-IT-0.2, ⑧ TiO_2_-IT-0.36, ⑨ TiO_2_-IT-1.0) are shown in Figure 2 in the same form. Each point in Figure 1 and Figure 2 indicates the amount of particles in each rat lung, and that is the sum of the amounts in the whole lung after BAL and in the BALF. The deposition fraction in each inhalation study was about 10%, and in the instillation studies, the amount at 3 days after the instillation was about 70% (63–83%) of the instilled amount.

The amounts of NiO and TiO_2_ in the lung decreased exponentially in both the inhalation and instillation studies. Each point is approximated as a one-compartment model, and the fitted lines are described in the figures. The biological half times (BHTs) were calculated by the following Formula (1) and (2):

M = M0 × exp(−kt)(1)

T(1/2) = ln2/k(2)

M: amounts in lung; M0: amounts at just after the inhalation/instillation; k: clearance rate constant; t: time; T(1/2): biological half time (BHT).

The calculated BHTs of ① NiO-IH-L and ② NiO-IH-H in the inhalation studies were 2.9 and 5.2 months, respectively. As for TiO_2_, the BHT of ③ TiO_2_-IH-L and ④ TiO_2_-IH-H were 2.0 and 1.8 months, respectively. In the inhalation studies, the BHTs in both of the NiO inhalation groups were delayed compared with those in the TiO_2_ inhalation groups.

In the instillation studies, the BHT of ⑤ NiO-IT-0.2 and ⑥ NiO-IT-1.0 were 4.9 and 9.5 months, respectively. In the TiO_2_ exposure groups, the calculated BHT of ⑦ TiO_2_-IT-0.2, ⑧ TiO_2_-IT-0.36, and ⑨ TiO_2_-IT-1.0 were 1.8, 1.8, and 2.2 months, respectively. Similar to the inhalation studies, all the BHTs in the NiO instillation groups were rather longer than those in the TiO_2_ instillation groups.

#### 2.1.2. Dose–Response Relationship between Lung Burden and BHT

**Figure 3:**
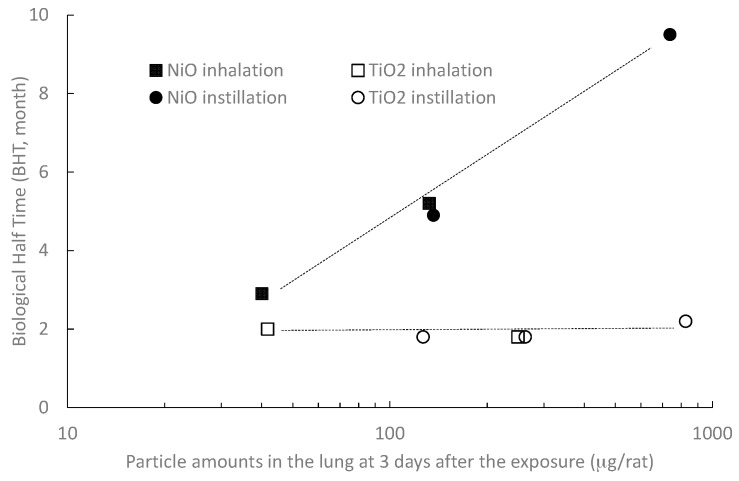
Relationship between lung burden and biological half time in inhalation and instillation studies.

When comparing the particle toxicities, the doses in the lung must be the same, because the lung response of inhaled particles depends on the dose and the toxicity of the particles. The dose is clear in instillation tests, but in inhalation tests the amounts of particles deposited in the lung are generally calculated by using the Multiple-Path Particle Dosimetry (MPPD) model or other simulation model. In this study, however, quantitative analysis of the nanoparticles in the lungs enabled us to identify the dose. The relationship between the lung burden and biopersistence (BHT) is shown in Figure 3, which compares the toxicity between NiO and TiO_2_ nanoparticles. The lung burden at three days after instillation and those after 4 weeks inhalation was plotted along the horizontal axis. The BHT values of the TiO_2_ nanoparticles inhaled or instilled in the present study were 1.8–2.2 months. In comparison with these values, all of the BHT values in the NiO inhalation and instillation were longer. Especially, although the amount of TiO_2_ in the lung at 3 days after the 1.0 mg instillation was almost the same as that in the NiO 1.0 mg instillation, the clearance delay in the NiO 1.0 mg instillation study was obvious. The BHTs in the NiO exposure groups were longer than those at the same TiO_2_ nanoparticles burden, and increased in a dose-dependent manner.

### 2.2. Cells in Bronchoalveolar Lavage Fluid (BALF) after NiO and TiO_2_ Inhalation

**Figure 4:**
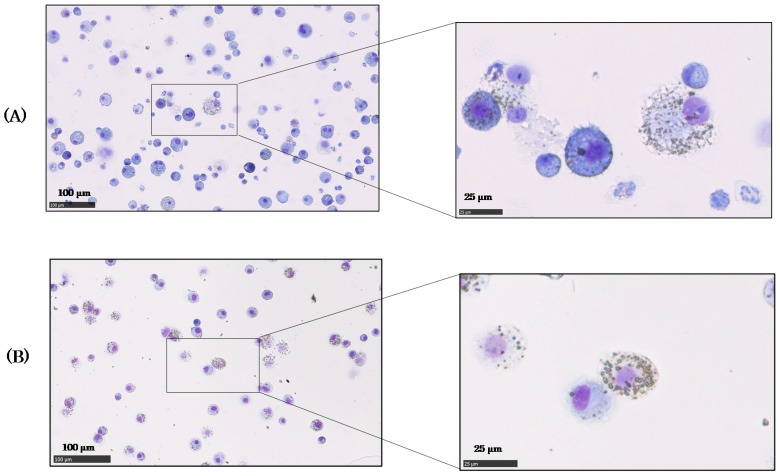
Cells in bronchoalveolar lavage fluid (BALF) at 3 days after the inhalation. (**A**) ② NiO-IH-H; (**B**) ⑥ TiO_2_-IH-H.

Light microscopic photos of cells in the BALF at 3 days after NiO and TiO_2_ inhalation at the high concentration are shown in Figure 4. Nanoparticles were phagocytized by macrophages in both studies, and each particle seemed to exist individually inside the macrophage. However, many polymorphonuclear neutrophils (PMN) cells indicating inflammation infiltrated into the BALF exposed to NiO, and some cells that engulfed NiO particles expanded and burst. Cells with TiO_2_ particles were almost normal. 

### 2.3. Histopathological Finding in the Lungs

Histopathological photos of lung at 3 days after inhalation and at 3 months after instillation of each type of nanoparticle are shown in the Supplementary File.

A mild infiltration of alveolar macrophages and neutrophils in the alveoli and interstitial area was observed in the NiO exposure group following inhalation at 3 days and at 1 month after exposure. In the TiO_2_ exposure group, some alveolar macrophages with a pigment-like material deposition were observed in the alveoli at 3 days after exposure (Figure S1).

In the NiO 1 mg intratracheal instillation group, mainly neutrophil and macrophage infiltration into the alveolar space was found dose-dependently at 3 days post-exposure. Inflammatory cells infiltrated into the subpleural space, and foamy and enlarged macrophages were often found in the alveolar space at 3 months post-exposure. In the TiO_2_ 1 mg exposure group, intra-alveolar infiltration of neutrophils was observed at 3 days and at 1 week post-exposure, and disappeared at 3 months post-exposure (Figure S2).

### 2.4. The Relation between BHT and the Other Indicator

**Figure 5:**
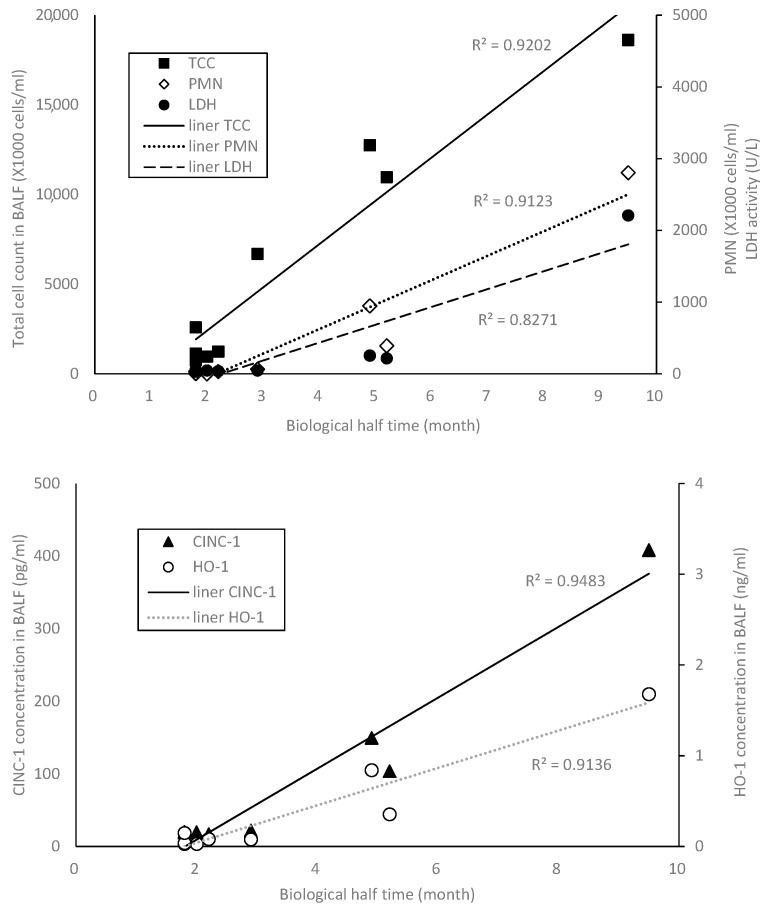
Relationship between biological half time and the other biomarkers. (Mean total cell count (TTC), polymorphonuclear neutrophils (PMN), lactate dehydrogenase (LDH), chemokine-induced neutrophil chemoattractant (CINC-1), and hemo oxygenase-1 (HO-1) at 1 month after the exposure).

A study by Morimoto et al. [8], in which the experimental conditions were the same as in the present study, reported increased lactate dehydrogenase (LDH), chemokine-induced neutrophil chemoattractant (CINC-1) and hemo oxygenase-1 (HO-1) related to oxidative stress in BALF. Figure 5 shows the relationship between BHT in the present study and the other biomarkers in their report. Each biomarker in Figure 5 is mean values at 1 month after the exposure because of a concern about bolus effects at 3 days and at 1 week after instillation. There are comparatively good correlations between BHT and total cell counts (TTCs), PMN, LDH, CINC-1, and HO-1 in the BALF. 

## 3. Discussion

We used two types of nanoparticles in this study—namely, high-toxicity NiO nanoparticles and low-toxicity TiO_2_ nanoparticles—in order to accurately determine their biopersistence and to investigate whether the biopersistence reflects the toxicity and can be used as a hazard indicator of nanoparticles.

Generally, particles that enter the lung are engulfed by scavenger cells like macrophages and cleared from the lung smoothly, but when particles have some toxicity, macrophages are injured and sometimes die. That impairs the alveolar macrophage-mediated clearance function, causing continued accumulation of the lung burden and prolonged biological half time. Biopersistence has been reported as a toxicity index for micron-size particles. For low-toxicity particles, the BHT values have been reported in the range of 60 to 80 days [11,33,34]. In the present inhalation study, the biological half times (BHTs) of NiO nanoparticles in high and low exposure concentrations were 5.2 months and 2.9 months, respectively, as shown in Figure 3. In comparison, the BHTs of TiO_2_ nanoparticles were 1.8 months in the high exposure concentration and 2.0 months in the low exposure concentration, which were at a roughly similar rate to micron-size particles, regardless of the amount of nanoparticles in the lungs. In comparison with the BHTs of TiO_2_, the NiO nanoparticles persisted in the lung, and the BHTs of inhaled NiO were prolonged, depending on the amount of particles in the lung. NiO nanoparticles reportedly elicit persistent pulmonary inflammation [35,36], and thus they are considered to be highly toxic, while TiO_2_ nanoparticles are thought to have low toxicity because of their transient effect on the lung [37,38]. The cells in BALF shown in Figure 4 also show an increased number of inflammation cells and some expanded or burst macrophages in the NiO group, in spite of having a lower particle burden than the TiO_2_ group. This also indicates that NiO nanoparticles are more toxic for macrophages or the other scavenger cells than TiO_2_ nanoparticles. In the histopathological observation shown in the Supplementary File, NiO exposure leads to infiltration of neutrophils and macrophages into the alveoli or interstitial tissue at 3 days after inhalation, but in the TiO_2_ inhalation group, pigment-like particles were observed in the alveoli. This indicates that the BHTs of inhaled NiO nanoparticles with high toxicity were longer than those of the TiO_2_ nanoparticles with low toxicity, and the longer BHT correlated with the toxicity of the particles.

In the instillation studies with the NiO nanoparticles, the BHTs of the 0.2 mg and 1 mg instillation groups were 4.9 months and 9.5 months, respectively. With the TiO_2_ nanoparticles, the BHTs of the 0.2 mg, 0.36 mg, and 1 mg instillation groups were 1.8 months, 1.8 months, and 2.2 months, respectively. Additionally, in the instillation studies, the BHTs of the TiO_2_ groups were around 2 months, which was similar to the inhalation studies. In comparison, the BHTs of the NiO instillation group were rather longer than those of the TiO_2_ groups. In the histopathological observation, the infiltration of neutrophils in the TiO_2_ 1.0 mg group was observed only for a short time after the instillation, while in the NiO instillation group these infiltrations were dose-dependent and continued to 3 months. The BHT results obtained in these instillation studies also reflect these pathological changes, and were prolonged when these inflammations were severe and persistent.

The BHTs of the TiO_2_ nanoparticles in both the inhalation and instillation studies were almost 2 months, which was the same as with micron-size particles. On the other hand, all the BHTs in the NiO exposure groups—both in inhalation and instillation—were longer than those of TiO_2_ nanoparticles in a dose-dependent manner (Figure 3). As the lung response to inhaled particles depends on the dose and the toxicity of the particles, a comparison of the BHTs of NiO and TiO_2_ nanoparticles at the same lung burden clearly shows that toxic NiO nanoparticles have a longer BHT, suggesting that biopersistence has the potential to be a good hazard indicator for inhaled nanoparticles.

Instillation studies have the problem that the lung burden with a large quantity of particles—albeit with low toxicity—can result in an excessive dose to macrophages, which would induce impaired clearance, accumulation of lung burden, and pathological changes [18,27,33,34]. In this study, the maximum dose of instilled nanoparticles we chose was 1 mg. The clearance of intratracheally instilled 1 mg TiO_2_ nanoparticles was not delayed (BHT: 2.2 months), suggesting that the volume of 1 mg nanoparticles (about 0.25 μL as volume calculated using the specific gravity of TiO_2_) is not an excessive level for rats. The volume of 1 mg NiO nanoparticles (about 0.15 μL also calculated using the specific gravity) is smaller than 1 mg TiO_2_ nanoparticles, indicating that the reason for the delayed BHT in the NiO 1 mg instillation was caused by the toxicity of the NiO nanoparticles, rather than an overloaded volume. Thus, the 1 mg used in this instillation study is thought to be an appropriate dose.

Comparing the inhalation and instillation methods, the amount of NiO in the lung at 3 days after 4 weeks of inhalation at the high concentration and that after the 0.2 mg instillation were almost the same. Similarly, the amount of TiO_2_ in the lung at 3 days after inhalation at the high concentration and that after the 0.36 mg instillation were almost the same. Although a single high dose of particles is known to induce delayed clearance due to the bolus effect [27,37], instillation studies result in almost the same BHT as in inhalation studies, probably because the nanoparticles are distributed evenly after instillation, causing a minimum bolus effect. Alternatively, the bolus effect after the intratracheal instillation might have been similar to the effect caused during the 4-week inhalation periods.

We performed inhalation and instillation studies and concluded that biopersistence—which reflected the histopathological changes, inflammatory responses, and the other biomarkers in BALF after the exposure to nanoparticles—will be a good hazard indicator in both inhalation and instillation studies, similarly to micron-size particles. The examination in this study was limited to the lungs, but the translocation of inhaled nanoparticles to other organs is also crucial to the evaluation of the biological effects of nanoparticles.

## 4. Materials and Methods

### 4.1. Inhalation and Intratracheal Instillation Studies

Inhalation and intratracheal instillation methods of this animal studies have been reported previously in detail [8], as described briefly below. We added 0.36 mg to the TiO_2_ instillation study. The experimental conditions are summarized in Table 2.

#### 4.1.1. NiO and TiO_2_ Nanoparticles

NiO (US3355, US Research Nanomaterials, Houston, TX, USA) and TiO_2_ (MT-150AW, Tayca Co., Ltd., Osaka, Japan) were individually dispersed in deionized water. The average agglomerated particle sizes as measured by a dynamic light scattering (DLS) analyzer (Zetasizer Nano ZS, Malvern Instruments Ltd., Worcestershire, UK) were 59.7 nm for NiO and 44.9 nm for TiO_2_. The physicochemical properties and TEM (transmission electron microscope) photos of two nanoparticles are shown in Table 3. Calculated crystal sizes from XRD spectra of raw powder using conventional Scherrer’s equation are 15.0 nm for NiO and 13.5 nm for TiO_2_. Those values are compatible with the averaged primary particle sizes measured from TEM images for sample particles (19 nm for NiO and 12 nm × 55 nm for TiO_2_). This means that primary particles seem single crystal-like and nano-size sample particles used in this study show a good crystallinity.

#### 4.1.2. Animals

Three hundred and forty four male Fisher rats were purchased from Charles River Laboratories Japan, Inc. (Yokohama, Japan). All procedures and animal handling were done in accordance with the guidelines described in the Japanese Guide for the Care and Use of Laboratory Animals as approved by the Animal Care and Use Committee, University of Occupational and Environmental Health, Japan and the approval codes are AE11-012, AE12-004, AE12-005 (approved on 30 March 2012).

#### 4.1.3. Inhalation and Intratracheal Instillation Methods

In the inhalation studies, particles were aerosolized by a nebulizer [39], and the rats were exposed to NiO and TiO_2_ nanoparticles in an exposure chamber for 4 weeks (6 h/day, 5 day/week) from age 10 to 13 weeks. Control rats were exposed to fresh air. The exposure concentrations of NiO were 0.32 ± 0.07 (① NiO-IH-L) and 1.65 ± 0.20 (② NiO-IH-H) mg/m^3^, and the exposure concentrations of TiO_2_ were 0.50 ± 0.26 (③ TiO_2_-IH-L) and 1.84 ± 0.74 (④ TiO_2_-IH-H) mg/m^3^, and 10 rats from each exposure group were sacrificed at 3 days and at 1 and 3 months after the inhalation. In both the NiO and TiO_2_ studies, at each sacrifice time the five rats were treated by bronchoalveolar lavage (BAL) using a saline solution, and the whole lung after BAL and a part of the BAL fluid were used for the measurement of the amount of particles in the lung.

In the instillation study, NiO and TiO_2_ nanoparticles dispersed in 0.4 mL distilled water were intratracheally instilled in rats. The doses were 0.2 mg (⑤ NiO-IT-0.2) and 1.0 mg (⑥ NiO-IT-1.0) for NiO, and 0.2 mg (⑦ TiO_2_-IT-0.2), 0.36 mg (⑧ TiO_2_-IT-0.36), and 1.0 mg (⑨ TiO_2_-IT-1.0) for TiO_2_. Control rats were instilled with 0.4 mL distilled water only. At 3 days, 1 week, and 1, 3, and 6 months after the instillation, 10 rats from each exposure group were sacrificed and treated by the same methods as in the inhalation study.

### 4.2. Measurement Methods of Each Nanoparticle Amounts in Lung

**Figure 6:**
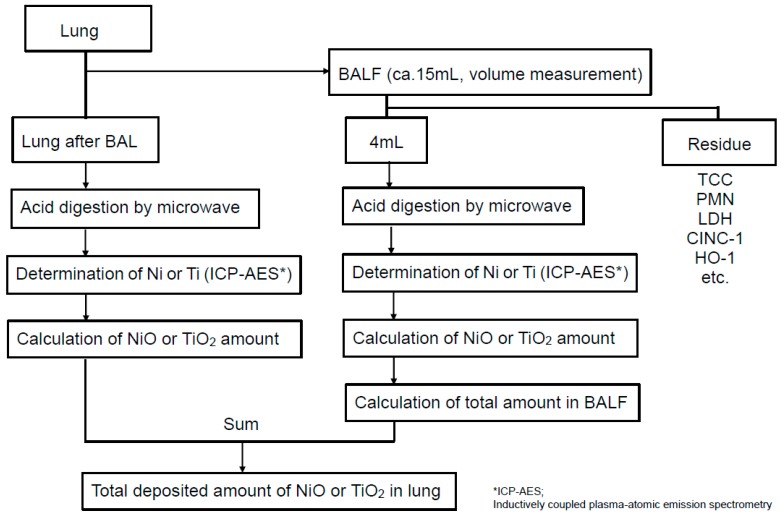
Determination method of NiO or TiO_2_ in lung.

Five rats were treated by bronchoalveolar lavage (BAL), and the amounts of particles in whole lung after BAL and in BALF were determined individually. The determination scheme is summarized in Figure 6.

For determination of NiO in lung, the lungs after BAL were digested with HNO_3_ and H_2_O_2_ in a Teflon flask at a high-temperature and high-pressure condition by a microwave digestion system (Ethos One system, Milestone, Italy) for 45 min. The microwave decomposition of organics with NiO was performed by controlling the temperature in the flask. Briefly, the temperature in the flask was gradually increased up to 50 °C, paused for a few min, then increased up to 180 °C and paused again and maintained at 180 °C for 10 min. The digestion program of NiO is shown in Table 4. The completely digested solutions were transferred to a volumetric flask, and the obtained constant volume and Ni content in the solution were determined by ICP-AES (Inductively Coupled Plasma-Atomic Emission Spectrometer SPS3500DD, SII NanoTechnology, Tokyo, Japan). The mass of NiO in each lung and the BALF were calculated from the determined amounts of Ni divided by the Ni content of the NiO. For the measurement of the amount of NiO in the BALF, the volume of BALF was measured after BAL, and the amount of particles in 4 mL of the fluid was determined by the same method as with the lung. The amount of particles in the BALF was calculated using the volume ratio. The total amount of NiO in the lung was the sum of the amounts in the whole lung after BAL and in BALF.

To determine the amounts of TiO_2_, the lungs after BAL and BALF were used in almost same way as in the determination of NiO, but the digestion reagents and program were different. For TiO_2_ analysis in the lung and BALF, HNO_3_, H_2_SO_4_, (NH_4_)_2_SO_4_, and H_2_O_2_ were used and digested for 30 min. In the digestion program for TiO_2_, the temperature was increased up to 240 °C and maintained for 20 min. The digestion program of TiO_2_ is also shown in Table 4. Similarly to NiO, the amount of Ti in the lungs and BALFs determined by ICP-AES was calculated from the amount of Ti in the digested solution divided by the Ti content of TiO_2_.

Before the determination, recovery tests were performed and the accuracy of the results was confirmed. The recovery rates for each material were over 95% in the lung and BALF. The determination limit was about 1 microgram for each sample. The amounts of NiO and TiO_2_ in the lungs of the controls that inhaled fresh air and were injected with distilled water were under the detection limit.

### 4.3. Observation of Cells in BALF

After measuring the particles, the rest of the BALF was used to observe the cells, which were collected by centrifugation (400× *g*, 15 min) and washed, and then splashed on a slide glass using cytospin. After that, the cells were fixed and stained with Diff-Quik (Systex Corp., Hyogo, Japan) for microscopic observation.

## 5. Conclusions

The usefulness of biopersistence as a hazard indicator of nanoparticles was investigated by examining the dose–response relationship of two nanoparticles (NiO and TiO_2_), each having a different toxicity, in inhalation and intratracheal instillation studies. After the exposure, the lung burden in each of five rats was determined by ICP-AES from 3 days to 3 months in the inhalation study and to 6 months in the instillation study. The calculated biological half times (BHTs) were almost the same in all the TiO_2_ nanoparticle exposure groups, but in the NiO nanoparticle exposure groups the BHTs were longer at the same burden and increased in a dose-dependent manner. These results indicate that biopersistence shown in BHTs is a good hazard indicator for nanoparticles.

## Figures and Tables

**Table 1:** Measured particle amounts in rat lungs.

Time	Measured Amounts of Nanoparticles in Rat Lungs (μg)														
	NiO						TiO_2_								
**Inhalation**															
	① NiO-IH-L			② NiO-IH-H			③ TiO_2_-IH-L			④ TiO_2_-IH-H					
3D	40.0	±	2.4	132.5	±	9.9	41.8	±	3.2	249.3	±	12.4			
1M	24.6	±	1.6	130.0	±	9.1	26.4	±	2.3	166.8	±	20.5			
3M	19.0	±	2.8	92.4	±	9.5	14.8	±	1.6	80.9	±	7.5			
**Instillation**															
	⑤ NiO-IT-0.2			⑥ NiO-IT-1.0			⑦ TiO_2_-IT-0.2			⑧ TiO_2_-IT-0.36			⑨ TiO_2_-IT-1.0		
3D	136.4	±	6.5	738.1	±	49.7	126.6	±	12.8	262.5	±	6.5	825.0	±	40.8
1W	128.7	±	15.8	645.9	±	194.5	130.0	±	6.1	240.6	±	24.4	835.5	±	29.4
1M	126.2	±	5.4	676.4	±	46.0	78.0	±	4.6	130.9	±	44.9	521.0	±	159.6
3M	95.6	±	13.9	539.5	±	119.1	31.2	±	1.6	53.8	±	10.6	278.5	±	80.0
6M	59.4	±	15.4	465.5	±	112.5	14.5	±	2.3	28.7	±	6.4	138.6	±	39.9

IH: inhalation study, IT: instillation study, L: exposure at low concentration, H: exposure at high concentration, Number: instilled amount, D: day, W: week, M: month.

**Table 2:** Experimental conditions.

Materials	NiO Nanoparticle		TiO_2_ Nanoparticle	
	US3355 (US Research Nanomaterials)		MT-150AW (Tayca Co., Ltd., Osaka, Japan)	
Whole body inhalation				
Exposure period	4 weeks (6 h/day, 5 days/week)		4 weeks (6 h/day, 5 days/week)	
Exposure concentration	① NiO-IH-L	0.32 ± 0.07 mg/m^3^	③ TiO_2_-IH-L	0.50 ± 0.26 mg/m^3^
	② NiO-IH-H	1.65 ± 0.20 mg/m^3^	④ TiO_2_-IH-H	1.84 ± 0.74 mg/m^3^
Sacrificed time	3 days, 1, 3 months after the inhalation		3 days, 1, 3 months after the inhalation	
Intratracheal instillation				
Instilled amount *	⑤ NiO-IT-0.2	0.2 mg	⑦ TiO_2_-IT-0.2	0.2 mg
	⑥ NiO-IT-1.0	1 mg	⑧ TiO_2_-IT-0.36	0.36 mg
			⑨ TiO_2_-IT-1.0	1 mg
Particle diameter (nm, DLS)	59.7 nm		44.9 nm	
Sacrificed time	3 days, 1 week, 1, 3, 6 months after the instillation		3 days, 1 week, 1, 3, 6 months after the instillation	

* mg/0.4 mL distilled water.

**Table 3:** Physicochemical properties of NiO and TiO_2_ nanoparticles.

Property	NiO Nanoparticle	TiO_2_ Nanoparticle
Shape *	Sphere	Spindle-shaped
Primary diameter *	19 nm	Short: 12 nm, Long: 55 nm
Purity *	More than 99.5%	99.5%
Surface area * (BET, m^2^/g)	57	111
Crystallinity (XRD spectra **)	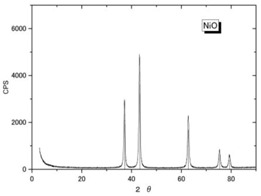	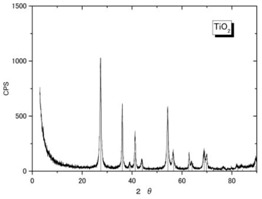
Size distribution (DLS)	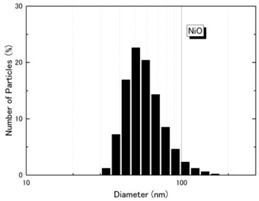	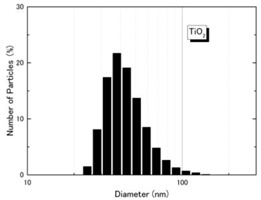
TEM picture	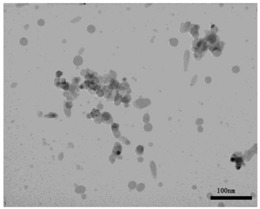	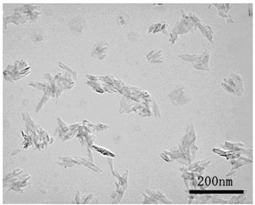

* Reference [8]. ** X-ray diffraction spectra (vertical line: reference data of NiO and rutile from JCPDS data-base). DLS: dynamic light scattering; TEM: transmission electron microscopy; XRD: X-ray diffraction.

**Table 4:** Digestion program of NiO and TiO_2_ nanoparticle in lung and BALF.

**NiO Nanoparticle**			
**Step**	**Time (min)**	Power (W) *	**Temperature (°C)**
1	2	1000	50
2	3	0	30
3	25	1000	180
4	1	0	150
5	4	1000	180
6	10	1000	180
**TiO_2_ Nanoparticle**			
**Step**	**Time (min)**	Power (W) *	**Temperature (°C)**
1	10	1000	240
2	20	1000	240

* Power is controlled automatically along the target temperature.

## Supplementary Materials

Supplementary materials can be found at www.mdpi.com/1422-0067/18/12/2757/s1.
