# Supplementary material for: Biopersistence of NiO and TiO2 Nanoparticles Following Intratracheal Instillation and Inhalation
Source: Int J Mol Sci. 2017 Dec 19;18(12):2757. doi: 10.3390/ijms18122757 (PMC5751356; doi:10.3390/ijms18122757)
Supplement: Supplementary file 1 [file ijms-18-02757-s001.pdf]

Supplementary Materials:

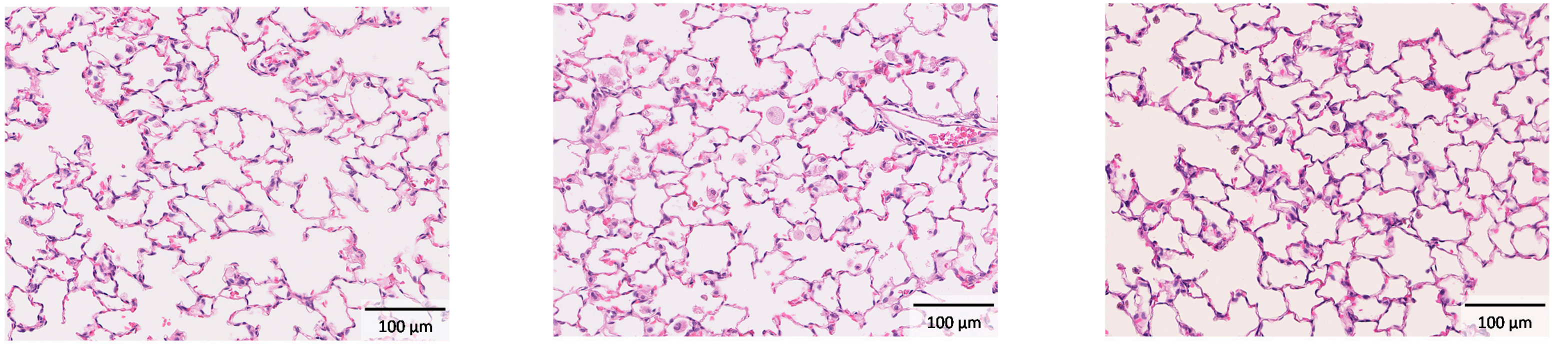

Control group NiO exposure group TiO_2 exposure group

Figure S1. Histopathological findings in the lungs at 3 days after inhalation (high concentration exposure).

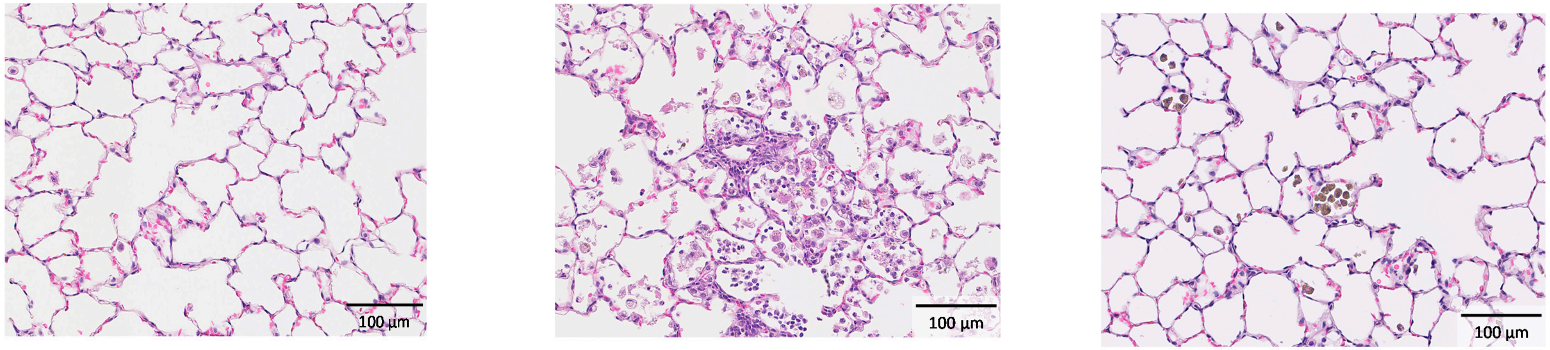

Control group NiO exposure group TiO_2 exposure group

Figure S2. Histopathological findings in the lungs at 3 months after instillation (1 mg instillation).
